# Bronchocele Cured by the Hypodermic Injection of Tincture of Iodine

**Published:** 1882-11-20

**Authors:** O. W. Schindel

**Affiliations:** Cumberland, Md.


### BRONCHOCELE CURED BY THE HYPODERMIC INJECTION OF TINCT. IODINE.

By O. W. SCHINDEL, M. D., CUMBETLAND, MD.

The following case shows how rapidly and radically some cases of bronchocele may be cured by the injection of iodine into the substance of the gland without any bad results or discomfort to the patient.

Mr. H., a lawyer by profession, aged 40 years, formerly of Cumberland, but now of Baltimore city, came to me in June, 1879, with a bronchocele as large as a good sized fist, situated immediately over the trachea. The tumour occupied the isthmus of the thyroid gland more particularly, but also extended into either wing. There was goitre in the patient's family on the mother's side. The enlargement of the neck was first noticed about eighteen months previous to my seeing the case; it was increasing much more rapidly he thought of late. The patient complained of a constriction about the fauces, oppression of chest with difficult breathing and an impending sense of suffocation which was increased when he lay down. From the firmness of the tumour and its general feel it consisted merely of hypertrophied glandular tissue.

Previous to his coming under my care he had been treated by another physician, for some months, by internal and local medication, but without any perceptible benefit or diminution in the size of the tumor.

On the 1st day of July, 1879, I injected (with an ordinary hypodermic syringe) deep into the gland forty minims of tinct. iodine of the following strength: officinal tinct. iodine, three parts, alcohol, one part. The operation did not cause him any discomfort or the slightest inconvenience. There was a sensation of warmth complained of in the gland for a few minutes, but this soon passed off and at the end of a half hour the patient walked to his home some distance from my office. On the 10th I repeated the operation with as much comfort. The tumor had diminished rapidly in size after each injection, and on the 3d day of August, a little more than one month from the first operation, I made the third and last injection, which entirely cured the patient.

I have frequently examined the case in the last two and a half years, and there only remains a small indurated nodule about the size of a bean, which can only be detected by carefully pinching up the tissues. In making the injection I was careful to avoid any cutaneous veins, and after thrusting the needle in the required depth to withdraw it slightly so as to disengage its point from any of the deeper veins.

This case demonstrates the fact that a strong solution of iodine may be thrown with impunity into hypertrophied thyroid tissue when the patient's general health is good without setting up any inflammatory action, and by this means curing that unsightly trouble commonly known as goitre, when it resists local and internal medication. When the proper precautions are taken, and there is nothing in the patient's condition to contraindicate this procedure, I deem it a perfectly safe and a rapid means of cure.

